# Relative Age and Maturity Timing in Youth Team Sports: Associations with Body Size and Vertical Jump Performance

**DOI:** 10.3390/sports14090365

**Published:** 2026-08-24

**Authors:** Cíntia França, Francisco Teixeira, Francisco Martins, Honorato Sousa, Koulla Parpa, Fahri Safa Cinarli, Élvio R. Gouveia

**Affiliations:** 1 Department of Physical Education and Sport, University of Madeira, 9020-105 Funchal, Portugal; 2 LARSyS, Interactive Technologies Institute, 9020-105 Funchal, Portugal; 3 The Interdisciplinary Center for the Study of Human Performance (CIPER), Faculty of Sport Sciences and Physical Education, University of Coimbra, 3004-504 Coimbra, Portugal; 4 Research Center in Sports Sciences, Health Sciences and Human Development (CIDESD), 5001-801 Vila Real, Portugal; 5 School of Science, University of Central Lancashire Cyprus (UCLan Cyprus), Pyla 7080, Cyprus; 6 Faculty of Sport Science, Inonu University, Malatya 44280, Türkiye; 7 The Interdisciplinary Center for the Study of Human Performance (CIPER), Faculty of Human Kinetics, University of Lisbon, Cruz-Quebrada, 1649-004 Lisbon, Portugal; 8 Swiss Center of Expertise in Life Course Research LIVES, 1227 Carouge, Switzerland

**Keywords:** relative age effect, biological maturation, muscle power, youth sport

## Abstract

Relative age effect (RAE) and biological maturation can significantly impact athlete selection. This study examined the presence of RAE in youth team invasion sports and assessed whether the timing of maturity influences body size and lower-body explosive strength. Athletes were grouped by age category (U14, U16, U18), and the distribution of birth quartiles was analyzed. Maturity timing was estimated using age at peak height velocity (APHV), and lower-body explosive strength was evaluated through jumping performance. Comparisons across age groups showed progressive increases in body size and jumping performance with advancing age, consistent with pubertal changes in fat-free mass and neuromuscular function. The birthdate distribution indicated an overrepresentation of relatively older athletes (U16 and U18), suggesting that selection bias may intensify with age around the pubertal window. Regression analyses showed that earlier APHV was significantly associated with greater body size after controlling for chronological age (CA) (*p* ≤ 0.01), although CA remained the strongest predictor. In contrast, APHV did not significantly explain additional variance in jumping performance, suggesting that anthropometrics, training exposure, and movement technique likely contributed. The current findings underscore the role of biological maturation in body size and the need for youth sport systems to include structured strength and conditioning programs that optimize lower-body muscle power.

## 1. Introduction

Worldwide, youth team sports systems are typically organized around annual age-grouping, in which individuals are classified by chronological age (CA) cutoffs. These grouping systems inherently produce relative age differences between those born early in the selection year (e.g., January) and those born later (e.g., December) [1]. A relative age effect (RAE), in which athletes born earlier in the selection year are overrepresented in elite youth sports compared to their younger peers, has been reported across multiple youth sports contexts, including basketball [2,3], football [4,5], and handball [6]. The primary consequence of RAE is a systematic selection bias that affects talent identification processes [7,8], often affording older athletes’ greater access to high-level coaching, competitive opportunities, and development resources. In contrast, increased dropout rates are observed among relatively younger athletes [2,9,10]. Importantly, it is of note that RAE is a result of date of birth, independent of an individual’s biological development.

Distinct from relative age, biological maturation is an independent physiological construct characterized by progress toward a fully mature state, with substantial variation among individuals in both timing and tempo [11]. Within a given CA cohort, significant inter-individual variability has been reported in somatic growth and biological development [12]. According to the literature, advanced biological maturity is frequently associated with temporary physical advantages, including larger body mass and stature, greater lean mass, and enhanced physical fitness attributes such as strength, muscular power, and speed [12]. Consequently, early-maturing athletes tend to be favored in selection procedures, regardless of their birth quartile [13,14]. Indeed, greater physicality and athleticism have remained crucial determinants in youth team sport selection processes [15].

To evaluate biological maturation without exposing young athletes to invasive or costly procedures, sport scientists and practitioners have heavily relied on non-invasive somatic estimation methods. For assessing maturity status and timing, the percentage of predicted adult height and maturity offset methods have been widely used in youth sports contexts [16]. The percentage of predicted adult height method estimates an individual’s adult height and expresses their current height as a percentage of that predicted height [17]. On the other hand, the maturity offset method estimates the number of years an individual is before or after peak height velocity (PHV), which can then be used to estimate age at PHV [18]. In estimation methods, some limitations on accuracy have been described, particularly the tendency to overestimate the timing of PHV in earlier-maturing athletes and to underestimate it in later-maturing peers [19]. However, given the invasiveness and high cost of gold-standard procedures, the literature has recommended non-invasive methods as suitable and practical alternatives for assessing maturity status and the timing of PHV [13].

The previous literature investigating the interactions between RAE and biological maturation has concluded that body size and physical performance are related to players’ maturity status, but not to their relative age [20,21]. However, most research on this topic has focused on general physical performance, such as sprint, change-of-direction, and jumping abilities, within a single-sport cohort [20,21,22].

Therefore, the current study aims to examine the independent effects of relative age and biological maturation on body size and lower-body muscle power, which are considered crucial to sport performance, across youth athletes from different team sports. Specifically, it was hypothesized that significant differences in body size and lower-body muscle performance would be observed with respect to relative age and biological maturation. In addition, consistent with the previous literature, it was hypothesized that athletes’ maturity status would contribute significantly to body size and lower-body muscle power performance, independently of relative age.

## 2. Materials and Methods

### 2.1. Study Design

All athletes were evaluated within a single month at the beginning of the sports season. The evaluation took place in a laboratory setting during the morning, with groups of 18 to 20 individuals. Participants were instructed to use their usual training clothes and shoes during data collection and to fast upon arrival at the laboratory. First, height and body composition were assessed in fasted conditions. Following these baseline assessments, participants were given a 15 min break to eat a light snack before completing the lower-body muscle power tests. All participants were free of injuries at the time of data collection.

### 2.2. Participants

A total of 587 male youth athletes (age = 15.3 ± 1.5 years; body mass = 61.6 ± 10.9 kg; stature = 171.3 ± 9.1 cm) were recruited from local sport clubs and included in the study. In total, 490 athletes played football, 46 played basketball, and 51 played handball. All participants were actively engaged in their sport, with at least three training sessions per week.

Sample size analysis was conducted using G*Power 3.1 [23]. For the group comparisons, a one-way analysis of variance (ANOVA) assuming an effect size of 0.25, 95% power, and a significance level of 0.05 indicated a required total sample of 252 participants for the three-group comparison (age groups) and 280 participants for the four-group comparison (birth quartiles). For the association analyses, a hierarchical multiple regression (fixed model, R^2^ increase) assuming an effect size of 0.15, 95% power, and α = 0.05 indicated a required total sample size of 89 participants with two predictors (chronological age and age at peak height velocity) to examine their influence on body size and lower-body muscle power outcomes.

All procedures implemented in this study were approved by the Ethics Committee of the University of Madeira (151/CEUMA/2024), and written informed consent to participate was obtained from all individuals and their respective legal guardians.

### 2.3. Anthropometry and Body Composition

Height was measured to the nearest 0.1 cm using a stadiometer (SECA 213, Hamburg, Germany). Following the International Society for the Advancement of Kinanthropometry (ISAK) protocol, participants stood barefoot, feet together, with their heads aligned in the Frankfort plane, and were required to draw in a breath while the investigator used a decompression technique to lift the head. Body composition was evaluated through hand-to-foot bioelectrical impedance analysis (InBody 770, Cerritos, CA, USA). All measurements were conducted in the early morning under standardized conditions, following an overnight fast. For the assessment, participants were barefoot and wearing only their underwear.

### 2.4. Biological Maturation

Chronological age (CA) was calculated to the nearest 0.1 years by subtracting the assessment date from the birth date. Maturity status was estimated using the maturity offset (MO) based on anthropometric measures [24], which has been reported to provide a close estimate of actual age at PHV [25]. The following equations were used to estimate time to/from the PHV:

$$maturity\;offset\;\left(boys\right)=\;-7.999994\;+(0.0036124\times \left(CA\;\times \;Height\right))$$

The age at peak height velocity (APHV) was then estimated as the difference between CA and the MO [26,27].

### 2.5. Birth Quartile

Participants’ dates of birth were divided into four equal 3-month quartiles [28,29]. Athletes born during the months of January, February, and March were framed into the first quartile (Q1); athletes born during April, May, and June composed the second quartile (Q2); athletes born in July, August, and September made up the third quartile (Q3); and athletes born during October, November, and December belonged to the fourth quartile (Q4).

### 2.6. Lower-Body Muscle Power

Lower-body muscle power was examined using the countermovement jump (CMJ) and the squat jump (SJ). Four data-collection trials, separated by a 30 s rest interval, were conducted using the Optojump Next system (Microgate, Bolzano, Italy) for analysis and measurement. In both tests, participants had their hands on their waists to avoid arm-swing interference. Athletes were encouraged to jump to maximum height and return to the starting position after each jump. The best trial from each test was used for analysis.

For the CMJ, participants began standing tall with feet shoulder-width to hip-width apart. They then lowered into a countermovement position to a depth of their choice, followed by a maximal vertical jump. Any removal of hands from their waist during the countermovement triggered a repeat trial.

The SJ protocol began with the participant in a squat at about 90° knee flexion. Participants held this position for three counts under the researcher’s guidance, then jumped. Trials were repeated if the participant performed a lower squatting movement than the standardized depth.

### 2.7. Statistical Analysis

Descriptive statistics are presented as means ± standard deviations. After checking for data normality, a one-way ANOVA was conducted to analyze differences between age groups (U14, U16, and U18) in CA, biological maturation, body size, and lower-body muscle power. Then, several ANOVAs were conducted to examine differences in the assessed variables across birth quartiles. Homogeneity of variance was confirmed using Levene’s test (*p* > 0.05). Effect size (η^2^) was reported and interpreted following Cohen’s guidelines [30]: ≤0.01 (small), ≤0.06 (medium), and ≤0.14 (large).

The chi-square test (χ^2^) was used to assess significant differences in distribution by birth quartile. Finally, hierarchical multiple regression analyses were performed to examine the associations between CA (entered in step 1) and APHV (entered in step 2) and height, body mass, CMJ, and SJ performance (dependent variables). Preliminary analyses were conducted to ensure that no violations of the assumptions of linearity, multicollinearity, or homoscedasticity occurred. Statistical analyses were conducted using IBM SPSS Statistics 31.0 (SPSS Inc., Chicago, IL, USA), and the significance level was set at 5%.

## 3. Results

Descriptive statistics for CA, biological maturation, body size, and lower-body muscle power across sport modalities are presented in Table 1. Mean CA and APHV values were slightly higher in the football group, which may be related to the significantly greater number of athletes in the older age categories (U16 and U18) compared with other team sports. Regarding body size, basketball athletes were taller (173.9 ± 10.7 cm), whereas handball athletes displayed the highest body mass (62.2 ± 16.6 kg). For lower-body muscle power, football and basketball athletes demonstrated similar vertical jump performance in both the CMJ (31.1 ± 5.2 cm and 30.6 ± 5.7 cm) and SJ (30.6 ± 6.5 cm and 30.3 ± 5.4 cm), whereas handball athletes recorded lower values across both jump tests.

Descriptive statistics and between-group comparison across age groups are summarized in Table 2. Statistically significant differences were observed across all evaluated variables (*p* < 0.001), each demonstrating a medium to large effect size (η^2^ ≥ 0.10). A significant age-related increment was seen in body size measures (height: *F* = 123.4, *p* < 0.001, η^2^ = 0.30; body mass: *F* = 104.9, *p* < 0.001, η^2^ = 0.26). Lower-body muscle power performance results showed substantial improvement across age groups, with older athletes achieving higher performance levels than their younger counterparts.

**Figure 1:**
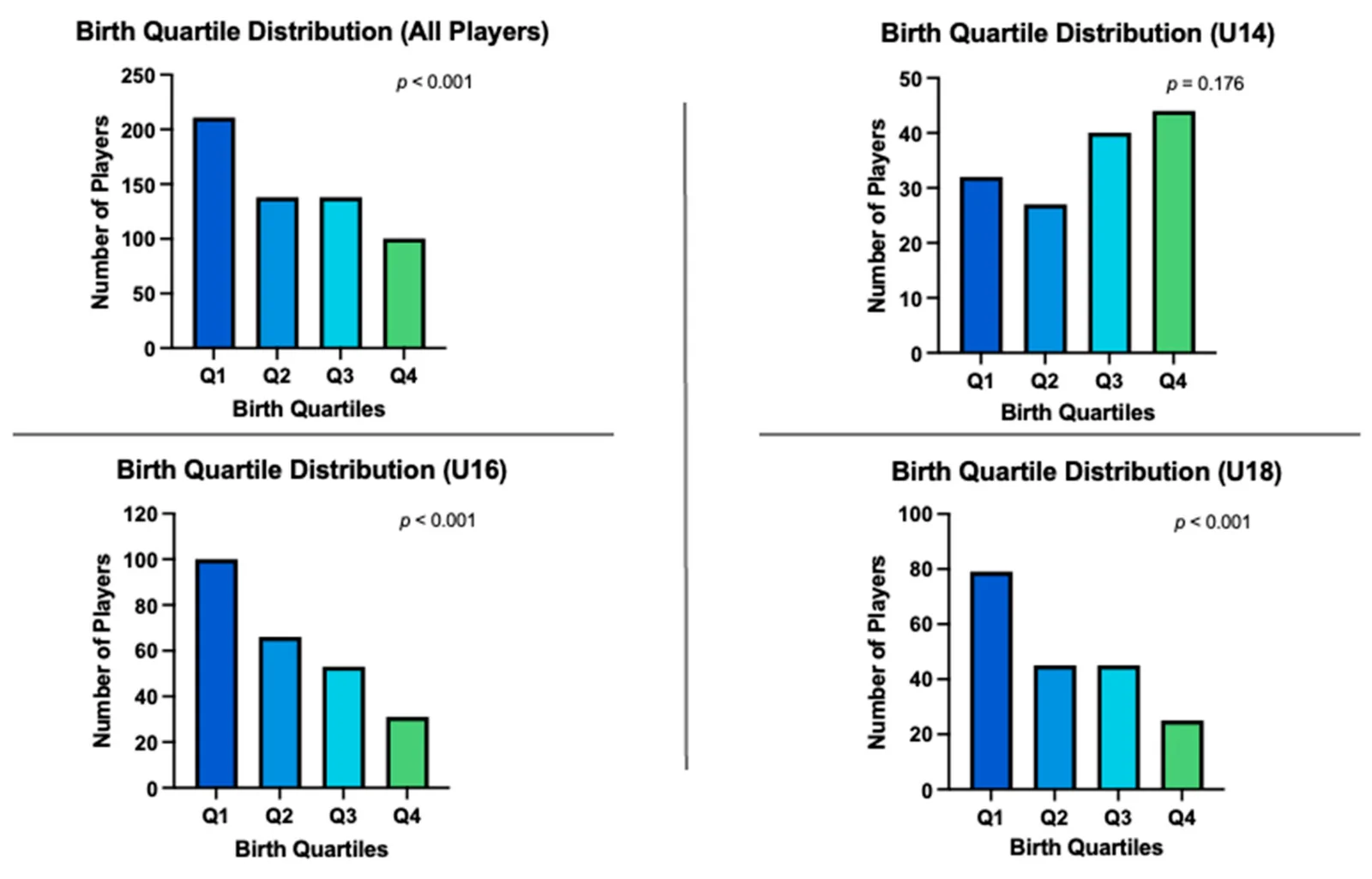
Birth quartile distribution across age groups.

The birth quartile (BQ) distribution is presented in Figure 1. Overall, a significant overrepresentation of athletes born in Q1 was observed (χ^2^ = 44.066, *p* < 0.001). The distribution revealed a prevalence of players born in Q1 and Q2 among the U16 and U18 groups (66.4% and 63.9%, respectively). Comparison results indicate a significant proportion of players born in Q1 in the U16 (χ^2^ = 40.016, *p* < 0.001) and U18 (χ^2^ = 31.072, *p* < 0.001) groups. In contrast, nearly 58.8% of the individuals in the U14 group belonged to Q3 and Q4; however, the differences were not statistically significant (χ^2^ = 4.944, *p* = 0.176).

Table 3 summarizes comparisons of CA, biological maturation, body size, and lower-body muscle power across birth quartiles. No statistically significant differences in maturity offset across BQ were observed in the U14 and U18 age groups (*p* = 0.351 and *p* = 0.099, respectively). On the contrary, a small but statistically significant difference was detected in maturity offset for the U16 age group (*F* = 3.783, *p* = 0.011, η^2^ = 0.04). Overall, no substantial differences were observed in BQ for body size and lower-body muscle power performance across age groups.

Results from hierarchical regression analyses predicting body size and lower-body muscle power from CA and APHV are presented in Table 4 and Table 5. The variance inflation factors (VIFs) and tolerance statistics confirmed that the regression models were free from multicollinearity.

CA was a strong positive predictor of height (β = 0.558, *p* ≤ 0.001), explaining 31.2% of the variance observed (R^2^ = 0.312; F = 264.684, *p* ≤ 0.001). APHV added a small contribution beyond CA in explaining variance in height, although it appeared as a significant negative predictor (β = −0.139, *p* ≤ 0.001). The same trend was observed for body mass, with CA emerging as a significant and positive predictor at step 1 (β = 0.549, *p* ≤ 0.001). Similar to height, adding APHV at step 2 explains only a small proportion of the variance in body mass (β = −0.117, *p* = 0.001).

When analyzing lower-body muscle power tasks, CA emerged as a significant and positive predictor of CMJ and SJ performance (β = 0.564, *p* ≤ 0.001; and β = 0.428, *p* ≤ 0.001, respectively). Adding APHV as a predictor did not significantly increase the model’s explanatory power for the CMJ and SJ results (β = −0.050, *p* = 0.158; and β = −0.050, *p* ≤ 0.138, respectively). Overall, the regression models explained 32.1% and 18.7% of the variance in CMJ and SJ performance.

## 4. Discussion

This study examined the independent effects of relative age and biological maturation on body size and lower-body muscle power performance in young team-sport athletes. The results indicate no significant differences in body size or lower-body muscle power performance by relative age. Independent of relative age, earlier APHV was associated with increased body size but not with lower-body muscle power performance.

Preliminary comparisons across age groups indicated a progressive and significant increase in body size and lower-body muscle power tasks with advancing age, as reported in the previous literature [31,32]. Overall, caution is recommended when interpreting the estimated APHV, as discrepancies have been reported between predicted and observed APHV, particularly with underestimation at younger ages and overestimation in older age groups [27,33].

When analyzing the distribution of birth quartiles, the results indicate a prevalence of players born in Q1, consistent with previous findings in different age categories of team sports [6,21,34,35]. The findings show that in the U16 and U18 groups, more than 63.5% of players were born in the first half of the year, while nearly 58.5% of players in the U14 were born in the second half of the year. Curiously, the number of athletes born in Q1 was substantially greater than in the other birth quartiles in the U16 and U18 age groups, which could initially suggest the presence of RAE. However, when comparing body size and lower-body muscle power performance among athletes from different birth quartiles, no significant differences were detected. On the contrary, substantial differences were found in APHV, with athletes born in Q1 showing earlier APHV than those born in Q4, especially in the U14 age group. Consistent with previous research, selection bias appears to favor biological maturation over relative age [36].

Meanwhile, the results of the hierarchical regression analyses indicate that earlier APHV was significantly associated with increased body size after controlling for CA. Thus, the timing of maturity still matters beyond CA, as players of the same age who mature earlier tend to be taller and heavier than their peers [11,37]. Nevertheless, CA remained the most significant predictor of the model, suggesting that body size is primarily age-dependent across adolescence, whereas maturity timing accounts for comparatively smaller within-age differences.

In contrast, APHV did not make a significant contribution to the variance observed in lower-body muscle power tasks. Although neuromuscular function tends to improve during puberty, leading to increased strength [38], vertical jumping may be influenced by multiple neuromuscular and mechanical factors. Previous research investigating factors associated with muscle strength in trained male adolescents concluded that the apparent influence of maturation on CMJ outcomes was primarily attributable to changes in body size and composition, particularly fat-free mass and lean leg volume [39]. In a recent study, a more efficient stretch-shortening cycle was observed in post-PHV individuals, suggesting that maturity status influences jump performance [40]. On the other hand, the literature has also reported that vertical jump height is determined by muscle work and efficacy [41,42] and by trainability [43]. These findings highlight that the timing of maturity contributes to anthropometric differences but does not independently determine explosive performance, underscoring the multifactorial nature of athletic development. Therefore, coaches and practitioners in youth sports should be aware that differences in lower-body muscle power tasks are shaped by both biological (growth-related changes) and environmental mechanisms (training exposure and movement technique), underscoring the importance of incorporating strength programs into training periodization.

The cross-sectional design used in this study is a limitation, as a longitudinal follow-up would allow for a more in-depth analysis of individual growth and developmental trajectories. Additionally, variables related to body composition, particularly fat-free mass and body fat, as well as training exposure, might play a significant role in lower-body muscle power performance [11,44], but they were not assessed in the current analysis. It is also important to recognize that the sample age range mostly captured athletes categorized as circa-PHV and post-PHV, with a few pre-PHV individuals. For that reason, the assessment of the biological maturation process was limited due to the absence of a full spectrum of maturing individuals.

Despite these limitations, the present results provide evidence of the contribution of biological maturation to increased body size, even after controlling for CA, although it did not significantly explain the variance in jumping tasks. Based on these findings, trainability and technique may be crucial factors for lower-body muscle power performance, underscoring the need to consider strength programs in youth sports. Future research using longitudinal designs that track youth athletes from pre-PHV through post-PHV can help clarify the long-term interactions among biological maturation, body size, and neuromuscular adaptations. Finally, coaches should be aware of selection bias toward early-maturing individuals due to temporary advantages, especially in body size, which underscores the importance of providing equal participation opportunities during adolescence.

## 5. Conclusions

This study evaluated the independent contributions of relative age and biological maturation to body size and lower-body explosive power among young team-sports athletes. Overall, the findings show that relative age does not directly influence body size or lower-body muscle power performance across U14, U16, and U18 age groups. Although an overrepresentation of relatively older athletes, born in Q1 and Q2, is evident, body size is primarily governed by biological maturity rather than CA. Earlier APHV is significantly associated with increased body size after controlling for CA. In contrast, APHV did not independently predict the variance observed in lower-body muscle power tasks (CMJ and SJ), underscoring the multifactorial nature of explosive tasks such as vertical jumping. Ultimately, biological maturation should be considered in youth sport systems to account for temporary differences in physical variables associated with advanced maturity. Subsequently, strength and conditioning programs should be integrated into youth training periodization to promote movement technique development and the efficacy of muscle work, thereby improving lower-body muscle power.

## Figures and Tables

**Table 1:** Descriptive statistics for CA, biological maturation, body size, and lower-body muscle power performance according to sport modality.

Variable	Football (*n* = 490)	Basketball (*n* = 46)	Handball (*n* = 51)
	Mean ± SD	Mean ± SD	Mean ± SD
CA (years)	15.4 ± 1.4	14.7 ± 1.1	14.3 ± 1.9
APHV (years)	13.8 ± 0.5	13.4 ± 0.4	13.5 ± 0.5
MO (years)	1.66 ± 1.19	1.28 ± 1.14	0.77 ± 1.70
Height (cm)	171.3 ± 8.5	173.9 ± 10.7	168.4 ± 12.6
Body mass (kg)	61.7 ± 9.3	60.6 ± 12.5	62.2 ± 16.6
CMJ height (cm)	31.1 ± 5.2	30.6 ± 5.7	28.0 ± 7.7
SJ height (cm)	30.6 ± 6.5	30.3 ± 5.4	27.8 ± 7.3

CA (chronological age), APHV (age at peak height velocity), MO (maturity offset), CMJ (countermovement jump), SJ (squat jump), SD (standard deviation).

**Table 2:** Descriptive statistics and comparison across age groups for CA, biological maturation, body size, and lower-body muscle power performance.

Variable	U14 (*n* = 143)	U16 (*n* = 250)	U18 (*n* = 194)	Group Comparison		
	Mean ± SD	Mean ± SD	Mean ± SD	*F*	*p*	η^2^
CA (years)	13.3 ± 0.7	15.1 ± 0.6	16.9 ± 0.6	1493.9	<0.001	0.84 (large)
APHV (years)	13.7 ± 0.4	13.7 ± 0.5	14.0 ± 0.5	32.267	<0.001	0.10 (medium)
MO (years)	−0.03 ± 1.00	1.32 ± 0.57	2.76 ± 0.50	700.7	<0.001	0.71 (large)
Height (cm)	162.7 ± 8.7	172.8 ± 7.7	175.5 ± 6.7	123.4	<0.001	0.30 (large)
Body mass (kg)	52.8 ± 10.4	61.9 ± 9.8	67.7 ± 7.7	104.9	<0.001	0.26 (large)
CMJ height (cm)	26.5 ± 5.7	30.6 ± 4.5	34.1 ± 4.5	105.8	<0.001	0.27 (large)
SJ height (cm)	26.6 ± 9.4	30.1 ± 4.6	33.4 ± 4.3	53.1	<0.001	0.15 (large)

CA (chronological age), APHV (age at peak height velocity), MO (maturity offset), CMJ (countermovement jump), SJ (squat jump), SD (standard deviation).

**Table 3:** Descriptive statistics and comparison across birth quartiles for CA, biological maturation, body size, and lower-body muscle power performance in young team-sports athletes.

	BQ1	BQ2	BQ3	BQ4	Group Comparison		
U14	*n* = 32	*n* = 27	*n* = 40	*n* = 44	F	*p*	η^2^
CA (years)	13.6 ± 0.5	13.5 ± 0.3	13.3 ± 0.6	13.0 ± 0.9	6.939	<0.001	0.13 (medium)
APHV (years)	13.6 ± 0.4	13.5 ± 0.4	13.6 ± 0.4	13.8 ± 0.4	3.823	0.011	0.08 (medium)
MO (years)	0.18 ± 0.72	−0.22 ± 0.62	−0.16 ± 0.9	0.04 ± 1.38	1.102	0.351	0.02 (medium)
Height (cm)	166.0 ± 8.5	161.5 ± 8.3	162.8 ± 8.2	161.1 ± 9.0	2.333	0.077	0.05 (medium)
Body mass (kg)	54.8 ± 8.6	53.3 ± 9.6	53.1 ± 11.1	50.9 ± 11.4	0.900	0.443	0.02 (medium)
CMJ height (cm)	27.7 ± 5.0	26.3 ± 5.1	26.8 ± 6.9	25.3 ± 5.2	1.164	0.326	0.03 (medium)
SJ height (cm)	29.7 ± 16.6	26.2 ± 5.2	26.5 ± 6.5	24.7 ± 5.1	1.857	0.140	0.04 (medium)
U16	*n* = 100	*n* = 66	*n* = 53	*n* = 31			
CA (years)	15.1 ± 0.6	14.9 ± 0.6	15.0 ± 0.6	15.4 ± 0.5	4.704	0.003	0.05 (medium)
APHV (years)	13.7± 0.5	13.6 ± 0.4	13.8 ± 0.5	13.6 ± 0.4	1.234	0.298	0.02 (medium)
MO (years)	1.38 ± 0.52	1.15 ± 0.58	1.28 ± 0.61	1.52 ± 0.57	3.783	0.011	0.04 (medium)
Height (cm)	173.2 ± 7.1	172.0 ± 8.9	173.6 ± 8.3	172.3 ± 6.3	0.560	0.642	0.01 (small)
Body mass (kg)	61.2 ± 9.2	61.4 ± 10.7	63.2 ± 10.6	63.1 ± 8.1	0.705	0.550	0.01 (small)
CMJ height (cm)	30.9 ± 4.3	30.5 ± 4.9	30.5 ± 4.3	30.4 ± 4.3	0.163	0.921	0.00
SJ height (cm)	30.4 ± 4.5	30.2 ± 5.0	29.7 ± 4.7	30.0 ± 3.9	0.303	0.823	0.00
U18	*n* = 79	*n* = 45	*n* = 45	*n* = 25			
CA (years)	17.0 ± 0.6	16.9 ± 0.5	16.9 ± 0.7	17.0 ± 0.5	0.731	0.535	0.01 (small)
APHV (years)	13.9 ± 0.5	14.1 ± 0.5	14.1 ± 0.5	14.1 ± 0.4	1.766	0.155	0.03 (medium)
MO (years)	2.86 ± 0.45	2.65 ± 0.42	2.69 ± 0.60	2.80 ± 0.55	2.124	0.099	0.03 (medium)
Height (cm)	175.4 ± 6.5	175.3 ± 5.6	174.5 ± 8.0	178.0 ± 6.6	1.518	0.211	0.02 (medium)
Body mass (kg)	67.8 ± 7.6	68.7 ± 8.0	66.6 ± 7.4	67.9 ± 8.4	0.570	0.635	0.01 (small)
CMJ height (cm)	33.8 ± 4.8	34.4 ± 4.1	34.6 ± 4.5	33.8 ± 4.0	0.402	0.752	0.01 (small)
SJ height (cm)	33.4 ± 4.4	33.7 ± 4.2	33.8 ± 4.4	32.4 ± 4.1	0.599	0.616	0.01 (small)

CA (chronological age), APHV (age at peak height velocity), MO (maturity offset), CMJ (countermovement jump), SJ (squat jump), BQ (birth quartile).

**Table 4:** Summary of hierarchical regression analysis with CA and APHV predicting height and body mass.

Variable	Height		Body mass	
	Model I	Model II	Model I	Model II
	β	β	β	β
CA	0.558 **	0.599 **	0.549 **	0.584 **
APHV		−0.139 **		−0.117 **
				
R^2^	0.312	0.329	0.302	0.314
*F* for change in R^2^	264.684 **	143.307 **	252.828 **	133.804 **

Model I: CA; Model II: CA + APHV. CA (chronological age), APHV (age at peak height velocity). ** *p* ≤ 0.01.

**Table 5:** Summary of hierarchical regression analysis with CA and APHV predicting lower-body muscle power tasks.

Variable	CMJ		SJ	
	Model I	Model II	Model I	Model II
	β	β	β	β
CA	0.564 **	0.579 **	0.428 **	0.446 **
APHV		−0.050		−0.058
				
R^2^	0.319	0.321	0.183	0.187
*F* for change in R^2^	273.549 **	138.004 **	131.467 **	66.974 **

Model I: CA; Model II: CA + APHV. CA (chronological age), APHV (age at peak height velocity), CMJ (countermovement jump), SJ (squat jump). ** *p* ≤ 0.01.

## Data Availability

Due to privacy and ethical restrictions, the data used in this study are available upon request to the corresponding author.
